# *Weissella*, a novel lactic acid bacteria isolated from wild Sumatran orangutans (*Pongo abelii*)

**DOI:** 10.14202/vetworld.2019.1060-1065

**Published:** 2019-07-18

**Authors:** Safika Safika, Wardinal Wardinal, Yulia Sari Ismail, Khairun Nisa, Wenny Novita Sari

**Affiliations:** 1 Department of Veterinary Infection Diseases and Veterinary Public Health, Faculty of Veterinary Medicine, IPB University, Jalan Agatis, IPB Dermaga, Bogor 16680, Indonesia; 2 Department of Biology, Education and Teaching Faculty, Ar-Raniry State Islamic University Banda Aceh 23111, Indonesia; 3 Department of Biology, Faculty of Mathematics and Sciences, Syiah Kuala University, Banda Aceh 23111, Indonesia; 4 Department of Mathematics and Applied Sciences, Syiah Kuala University, Darussalam, Banda Aceh 23111, Indonesia

**Keywords:** 16S rRNA, API 50 CHL, lactic acid bacteria, orangutan, *Pongo abelii*, *Weissella*

## Abstract

**Aims:**

This study aimed to isolate and identify lactic acid bacteria (LAB) in wild Sumatran orangutans to provide more information about LAB diversity derived from Sumatran orangutan feces.

**Materials and Methods:**

Fecal sampling from three female orangutans, around 35 years old, was carried out in the wild forest areas at the research station of Suaq Belimbing Gunung Leuser National Park located in the South Aceh district. Orangutan fecal samples were taken in the morning when the orangutans first defecated. The orangutans were above the tree, which is approximately 12-15 m from the ground where feces were found.

**Results:**

Fermentation testing using the API 50 CHL Kit showed that OUL4 isolates were identified as *Lactococcus lactis* ssp. *lactis* with an identity value of 73.5%. Homology analysis demonstrated that the OUL4 isolates have 93% similarity to *Weissella cibaria*, and phylogenetic trees constructed using Mega 7.0 also showed that OUL4 isolates are related to *W. cibaria*.

**Conclusion:**

These results show that there is a difference in identification between biochemical testing with API kits and molecular analyses on LAB isolates from wild Sumatran orangutans. Based on 16S rRNA gene homology, the OUL4 LAB isolates from wild Sumatran orangutans have 93% homology to *W. cibaria*.

## Introduction

Orangutans are the only great apes living in Asia, whereas their relatives, gorillas, chimpanzees, and bonobos, live in Africa. Currently, around 90% of the great ape species are in Indonesia and are only found in Sumatra and Kalimantan. Orangutans that live in Sumatra belong to Sumatran orangutan (*Pongo abelii*) and Tapanuli orangutan (*Pongo tapanuliensis*) whereas *Pongo pygmaeus* occupy lowland forests in Kalimantan [1]. The International Union for Conservation of Nature [2] Red List of Threatened Species has added Bornean orangutans into the endangered animals’ category and Sumatran orangutans into the critically endangered category. Many research studies have been conducted on orangutans, including assaying for lactic acid bacteria (LAB) in their digestive tracts. LAB are nonmotile, anaerobic Gram-positive bacteria and do not form spores; however, some are also aerotolerant with negative catalase activity. Carbohydrate fermentation produces a final product in the form of lactic acid [3]. In the digestive tract, LAB are non-pathogenic and play a role in providing their host a defense against pathogenic microbes by maintaining the balance of the intestinal ecosystem, producing antimicrobial compounds that can inhibit or kill other pathogenic bacteria, such as bacteriocins, and can also synthesize various types of antagonistic molecules [4]. LAB are also called lantibiotics because they contain modified post-translational amino acids, such as lanthionine (two alanines linked by sulfur), β-methyl-lanthionine, dehydroalanine, and dehydrobutyrine, which are short peptides (19-13 amino acids) and are active in Gram-positive bacteria [5]. In addition, LAB are also enzyme producers [6], such as *Lactobacillus*, which produce cellulase enzymes that help the digestive process. This enzyme can break down coarse fiber components which are difficult to digest [7].

LAB can also produce interesting molecules, including gamma-aminobutyrate, exopolysaccharides, fructooligosaccharides, short-chain fatty acids, conjugated linoleic acid, and selenoproteins. LAB are also known as probiotics and antioxidants; can contribute to anti-prostate cancer and cholesterol reduction; can act as immunomodulators; and can be anti-inflammatory or pro-inflammatory [5]. LAB consist of the genera *Lactobacillus*, *Lactococcus*, *Leuconostoc*, *Streptococcus*, *Enterococcus*, *Pediococcus*, *Melissococcus*, *Carnobacterium*, *Oenococcus*, *Tetragenococcus*, *Vagococcus*, and *Weissella* [7]. The genus *Weissella* was introduced by Collins *et al*. [8], and was previously classified as *Leuconostoc* and *Lactobacillus* genera [9]. The *Weissella* genus consists of *Weissella* *hellenica*, *Weissella paramesenteroides* (formerly *Leuconostoc paramesenteroides*), *Weissella* *confusa*, *Weissella* *kandleri*, *Weissella* *minor*, *Weissella* *halotolerans*, and *Weissella* *viridescens* species [10].

Studies on Sumatran orangutans, in captivity or nature reserves, found LAB colonies with Gram-positive cocci and basil bacteria. The characterization of LAB from Sumatran orangutans in high hill zoos with the 16S rRNA gene has also been carried out [11]. The results indicated that the LAB in Sumatran orangutans are closely related to the *Lactobacillus helveticus* IMAU50151 strain, with a homology of 89%. So far, LAB have been widely used as probiotics because of their ability to inhibit enteropathic bacteria. A previous study by Septiarini [12] showed that the antimicrobial activity of LAB from orangutan *P. pygmaeus* feces can inhibit enteropathic bacteria such as *Escherichia coli*, *Salmonella*, and *Shigella*.

LAB from wild orangutans could likely be used as a probiotic to stabilize the gut and suppress the growth and colonization of bacterial enteropathogens when introduced into semi-wild usually undergoes a rehabilitation orangutans process before release back into the wild. LAB research on wild Sumatran orangutans is still rare. Therefore, this study aims to isolate and identify LAB from wild Sumatran orangutans to provide more information about LAB diversity derived from their feces. This study identified LAB originating from wild orangutan feces, which can potentially be used as probiotics to maintain the balance of normal intestinal flora in semi-wild orangutans that will be released back into nature.

## Materials and Methods

### Ethical approval

This study obtained a permit and a written recommendation letter from Lembaga Ilmu Pengetahuan Indonesia (LIPI) and Kementerian Lingkungan Hidup dan Kehutanan (KLHK) Direktorat Jenderal Konservasi Sumber Daya Alam dan Ekosistem. Nomor: SK 100/KSDAE/SET/KSA. 2/3/2017.

### Sample and materials

Three female orangutans, approximately 35 years old, were used to sample their feces. Orangutans, who roam freely in this forest, live naturally without human intervention, in health management, food, vaccination, and the provision of worm medicine; fecal orangutan sampling was carried out in wild forest areas. Their food are fruits that are picked and eaten directly from trees, and the orangutans are approximately 12-15 m from the ground where feces are found.

The tools used in this study were as follows: Petri dishes (Normax Marinha Grande-Portugal), Erlenmeyer flask (Phyrex, New York, USA), test tubes (Phyrex New York, USA), test tube racks, object glasses, measuring cups (Phyrex New York, USA), Beaker glasses (Phyrex, New York, USA), microtubes, analytical scales (A&D), incubator (Memmert, Schwabach, Germany), binocular microscope (Kruess, German), autoclave (Labocon, Leicester, United Kingdom), centrifuge (Eppendorf, Hamburg, Germany), oven (Memmert, Schwabach, Germany), laminar airflow (Esco Micro Pte. Ltd, Upper Changi Singapore), colony counter, micropipette (Eppendorf, Hamburg, Germany), polymerase chain reaction (PCR) machine (BioRad, California, USA), DNA electrophoresis apparatus (BioRad, California, USA), Ultraviolet transilluminator, Gel Doc (BioRad, California, USA), Vortex (Eppendorf), hot plate (Torey Swedesboro, USA), refrigerator (Samsung), glove, power supply (BioRad California, USA), chamber and tray (BioRad, California, USA), aluminum foil, microwave oven (Panasonic, Jakarta, Indonesia label paper, tissue, and cotton.

The sample materials used in this study were isolates of LAB from wild Sumatran orangutan (*P. abelii*) feces at the Suaq Belimbing South Aceh research station. The media materials used in this study were sterile aquadest, agar (OXOID Ltd., Basingstoke, Hampshire, England), liquid De Man, Rogosa, and Sharpe (MRS) Broth (OXOID LTD., Basingstoke, Hampshire, England), nutrient broth (OXOID LTD., Basingstoke, England), 96% alcohol, gentian violet (Merck Darmstadt, German), lugol (Merck Darmstadt, German), safranin (Merck Darmstadt, German), Presto™ Mini gDNA Bacteria KIT (Biotech Ltd. New Taipei City, Taiwan), ethanol, ddH_2_O, PCR reaction buffer, DNA Polymerase (Bioline), dNTPs, MgCl_2_, DNase-free water, primers (forward and reverse), agarose, Gel red (Fermentas), parafilm, dye loading, DNA samples, 1 kb DNA marker (Fermentas), and dry ice.

### Sample collection

Samples of feces were taken at the research station of Suaq Belimbing Gunung Leuser National Park located in the South Aceh district. Fresh fecal samples (35 g) were taken from the forest after defecation from a tall tree, and 1 g was used in the dilution process to isolate bacteria. Orangutan feces were taken early in the morning as soon as the orangutan defecated. The researcher monitored and followed up the orangutans for 1 day before sampling to look for the orangutan’s nest point, noting the coordinates using Global Positioning System (GPS). The next day, the researcher reached that point to wait for the orangutans to defecate, and as soon as possible took a sample before the feces were swarmed by orangutan feces-eating insects.

Stool samples were taken aseptically and put into sterile plastic bottles, stored in a cool box, and taken to the laboratory for analysis. The collected stool samples were serially diluted at 10^−1^-10^−6^ using sterile peptone water. After dilution, as much as 1 ml was plated using the pour plate method on selective MRS agar (MRSA) sterile medium.

Isolated LAB colonies were then identified by Gram staining, catalase testing, and fermentation testing using the API 50 CHL kit (API Bio Merieux, France) after incubation at 30°C for 24-48 h. Data analysis was carried out to identify species using the Apiweb™ Version 1.2.1 software (apiweb.biomerieux.com).

### DNA extraction

DNA extraction was performed using the Geneaid Presto™ Mini gDNA Bacteria Kit (Geneaid Biotech Ltd. New Taipei City, Taiwan) according to the manufacturer’s instructions. Extraction buffer (200 μl) was added to the pellets and then resuspended by pipetting or vortexing. Next, 20 μl of proteinase K was added and incubated at 37°C for 30 min, and the tube was inverted every 10 min. Thereafter, 200 μl of buffer Gene Binding (GB) was added to the sample and incubated at 70°C for 10 min followed by the addition of 200 μl of absolute ethanol and lysed by a shaker. The sample was added to a column with a 2-ml tube and centrifuged at 14,000-16,000 g for 2 min. The precipitate was transferred to a new 2-ml tube.

To the column, 400 μl of W1 buffer was added and centrifuged at 14,000-16,000 g for 30 s, and the supernatant was discarded. Next, 600 μl of wash buffer was added, centrifuged at 14,000-16,000 g for 30 s, and the supernatant was discarded. The column was centrifuged again at 14,000-16,000 g and transferred to a new 1.5-ml tube. Elution buffer (30-50 μl), which had been heated, was carefully added to the middle of the column and incubated at room temperature for 3-5 min followed by centrifugation at 14,000-16,000 g for 1 min. The purified DNA was then used for PCR [13].

### DNA amplification

The process for amplification of the 16S rRNA gene in LAB-isolate DNA was carried out using the forward BacF primer (5’-AGA GTT TGA TCM TGG CTC AG-3’), which is complementary to the conserved regions in the bacterial domain, and the reverse UniB primer (5’-GGT TAC STT GTT ACG ACT T-3’), which is based on the universal sustainable area of the 16S rRNA gene of *E. coli*, resulting in an amplicon length of around 1500 base pairs. Both of these primers are general primers for bacteria. The PCR began with a 5 min denaturation at 95°C, followed by 35 cycles of: 1 min denaturation at 95°C, 1 min annealing at 50°C, and 2 min elongation at 72°C, with a final extension performed at 72°C for 8 min [14].

### Phylogenetic analysis

Sequencing of the 16S rRNA gene was carried out by Macrogen Inc. (Korea). Analysis of the sequencing results was performed using BLAST (database available at www.ncbi.nlm.nih.gov). Double alignments were performed using Clustal W software (http://www.clustal.org). The visualization of kinship was performed using a combination of phylogenetic trees from MEGA 7.0 software (www.megasoftware.net) and neighbor-joining plots with the maximum composite likelihood method [15].

## Results

### Isolation of LAB

In this research, MRS medium was used to isolate LAB. This medium is selective for LAB, which includes species from the following genera: *Lactobacillus*, *Streptococcus*, *Pediococcus*, and *Leuconostoc* [16]. Six isolates were found, but two isolates could not be re-cultured. This article characterizes one isolate, OUL4, and other isolates will be discussed in another article.

**Figure-1:**
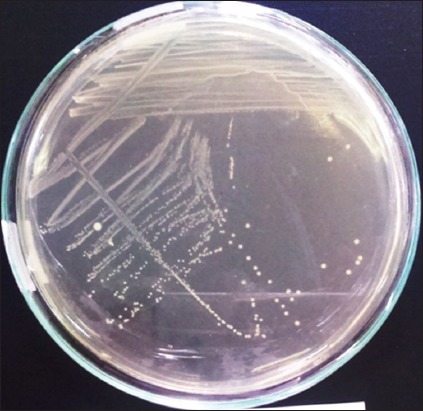
Colonies of lactic acid bacteria growing on De Man, Rogosa, and Sharpe agar media, incubation for 24 h.

**Figure-2:**
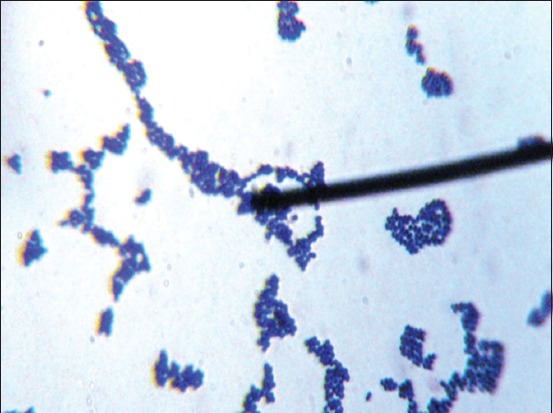
Gram staining of the lactic acid bacterial isolates from wild Sumatran orangutans with 1000x magnification.

The OUL4 colonies grown on MRSA media after incubation at 37°C were white, with circular form, entire edge, and convex elevation. Separate colonies were isolated using the scratch technique (Figure-1). Gram-positive staining revealed bacteria with a coccus cell shape (Figure-2), and they tested negative for catalase. Fermentation testing using the API 50 CHL Kit showed that the OUL4 isolates were identified as *Lactococcus lactis* ssp. *lactis* with an identity value of 73.5%. API 50 CHL media is a ready-to-use media containing 49 kinds of carbohydrates (Table-1). Classification of bacteria was based on the ability of isolated LAB to react with the carbohydrates used.

**Table 1:** Results of API 50 CHL Kit test LAB isolates from wild Sumatran orangutans.

No	Attributes	Sugar	Isolates OUL 4
0	0	*Control*	−
1	GLY	*Glycerol*	−
2	ERY	*Erythritol*	−
3	DARA	*D-Arabinose*	−
4	LARA	*L-arabinose*	−
5	RIB	*D-Ribose*	+
6	DXYL	*D-Xylose*	−
7	LXYL	*L-Xylose*	−
8	ADO	*D-Adonitol*	−
9	MDX	*Methil-ßD-Xylopyranoside*	−
10	GAL	*D-Galactose*	+
11	GLU	*D-Glucose*	+
12	FRU	*D-Fructose*	+
13	MNE	*D-Mannose*	+
14	SBE	*L-Sarbose*	−
15	RHA	*L-Rhamnose*	−
16	DUL	*Dulcitol*	−
17	INO	*Inositol*	−
18	MAN	*D-Mannitol*	−
19	SOR	*D-sorbitol*	−
20	MDM	*Methyl-αD-Mannopyranoside*	−
21	MDG	*Methyl-αD-Glucopyranoside*	−
22	NAG	*N-AcetylGlucosamine*	+
23	AMY	*Amygdaline*	−
24	ARB	*Arbutine*	+
25	ESC	*Esculine Ferric Citrate*	+
26	SAL	*Salicine*	+
27	CEL	*D-Cellobiose*	+
28	MAL	*D-Maltose*	+
29	LAC	*D-Lactose*	+
30	MEL	*D-Melibiose*	+
31	SAC	*D-Saccharose*	+
32	TRE	*D-Trehalose*	−
33	INU	*Inulin*	−
34	MLZ	*D-Melezitose*	−
35	RAF	*D-Raffinose*	−
36	AMD	*Amidon*	−
37	GLYG	*Glycogen*	−
38	XLT	*Xylitol*	−
39	GEN	*Gentiobiose*	+
40	TUR	*D-Turanose*	−
41	LYX	*D-Lyxose*	−
42	TAG	*D-Tagatose*	−
43	DFUC	*D-Fucose*	−
44	LFUC	*L-Fucose*	−
45	DARL	*D-Arabitol*	−
46	LARL	*L-Arabitol*	−
47	GNT	*Potassium gluconate*	-
48	2KG	*Potassium 2-Cetogluconate*	-
49	5KG	*Potassium 5-Cetogluconate*	-

LAB=Lactic acid bacteria

**Figure-3:**
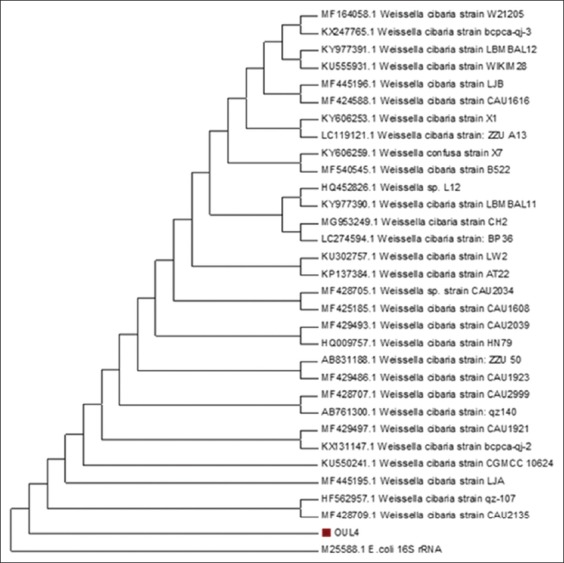
Phylogenetic dendrogram of the 16S rRNA sequence of the lactic acid bacterial isolates from wild Sumatran orangutans.

The results of homology analyses indicated that the OUL4 isolates have 93% similarity to *Weissella cibaria*, accession number MK377086 (Figure-3). Phylogenetic trees constructed using Mega 7.0 (www.megasoftware.net) also showed that the OUL4 isolates have a kinship with *W. cibaria* (Figure-3). The 16S rRNA gene has been well characterized with highly conserved domains. By employing PCR and sequencing analysis, this gene can be used to identify microorganisms and determine taxonomy, phylogeny, and species diversity [17]. Bosshard *et al*. [18] stated that an organism belongs to a specific species if it has a homology of ≥99%, and to a genus if it has a homology of ≥95-99%. In this study, the OUL4 isolates had 93% homology, indicating that the OUL4 isolates are likely a new species of the genus *Weissella*.

## Discussion

The results of the API 50 CHL KIT analysis identified the OUL4 isolates as *L. lactis* ssp. *lactis* with an identity level of 73.5%, and was contrary to the BLAST analysis, which showed homology to the genus *Weissella*. Previous research has also shown a difference between biochemical test results using API 50 CHL and molecular analyses [19]; we have also found contradicting results when comparing the API kit to molecular technique. While in this study, the results of molecular analysis were *Lactobacillus crispatus* (32.6%), *Lactobacillus jensenii* (25%), and *Lactobacillus gasseri* (20.6%), the results of the API 50 CHL Kit analysis found *Lactobacillus acidophilus* (34.8%), *L. crispatus* (27.2%), and *Lactobacillus fermentum* (13%). Furthermore, even though *L. acidophilus* had the greatest percentage found using the API 50 CHL, it was not detected by molecular testing.

The genus *Weissella* is comprised of a group of heterofermentative species, and belongs to the phylum Firmicutes, class Bacilli, order *Lactobacillales*, and family *Leuconostocaceae* [8]. They occur in pairs or in short chains, and some species have a tendency toward pleomorphism. They are facultative anaerobic chemoorganotrophs and are considered not to contain cytochromes. However, because many LAB have later been found to possess cytochromes and functional heme-dependent respiration, this may not be true in the case of *Weissella*. Glucose fermentation is heterofermentative, and carbohydrates are fermented via the hexose-monophosphate and phosphoketolase pathways. The end products of glucose fermentation are lactic acid, CO_2_, ethanol, and/or acetate. Depending on the species, the configuration of the lactic acid produced is either D-lactic acid or L-lactic acid. *Weissella* species generally require amino acids, peptides, fermentable carbohydrates, fatty acids, nucleic acids, and vitamins for growth. Biotin, nicotinic acid, thiamine, and pantothenic acid or its derivatives are required. Arginine is not hydrolyzed by all the species, and growth occurs at 15°C, while some species grow between 42°C and 45°C [20].

*Weissella* is a new genus, based on the analyses of 16S rRNA gene sequences and phylogenetic studies. The name *Weissella* is taken from Norbert Weiss, who focused his research on LAB, including the genus *Weissella* itself [8]. Currently, the *Weissella* genus consists of several species, including *Weissella beninensis* [21], *Weissella ceti* [22], *W. cibaria* [23], *W. confusa* [24], *Weissella fabaria* [25], *Weissella ghanensis* [26], *W. halotolerans*, *W. hellenica* [27], *W. kandleri*, *Weissella kimchii* [28], *Weissella koreensis* [29], *W. minor*, *W. paramesenteroides* [8], *Weissella soli* [30], *W. viridescens*, and *Weissella thailandensis* [31].

## Conclusion

The results revealed a difference in identification between the biochemical API kits and the molecular analyses of LAB isolates from wild Sumatran orangutans. Based on 16S rRNA gene analysis, the OUL4 LAB isolates from wild Sumatran orangutans have 93% homology to *W. cibaria*.

## Recommendations

Furthermore, this research should proceed to determine whether the isolate is a new species through certain analyses, i.e. DNA–DNA hybridization, %G+C content, and free fatty acid analysis.

## Authors’ Contributions

SS designed the study. WW collected and processed the samples for isolation and screening of cellulolytic bacteria. YSI and SS did DNA extraction and PCR. WW did phylogenetic analysis. KN and WNS interpreted the results and analyzed the data. All authors contributed equally in preparation and revision of the manuscript. All authors read and approved the final manuscript.
